# Local exchange of metabolites shapes immunity

**DOI:** 10.1111/imm.12978

**Published:** 2018-07-27

**Authors:** Felix Clemens Richter, Sandrine Obba, Anna Katharina Simon

**Affiliations:** ^1^ Kennedy Institute of Rheumatology Nuffield Department of Orthopaedics, Rheumatology and Musculoskeletal Sciences University of Oxford Oxford UK

**Keywords:** amino acids, bone marrow niche, fatty acids, haematopoiesis, immune cells, metabolism, stroma, vitamins

## Abstract

Immune cell differentiation and function depend on metabolic changes for the provision of energy and metabolites. Consequently, cellular metabolism relies on the availability of micronutrients such as vitamins and energy‐rich sources including amino acids and fatty acids. The bone marrow controls the continuous production of blood cells and is thereby reliant on the sophisticated interplay of progenitor and mature immune cells with its stromal microenvironment. The significance of stromal subsets in immunopoiesis is undisputed; however, our current knowledge is limited to their role in the production and secretion of a variety of soluble proteins such as cytokines. In contrast, the role of the haematopoietic niche in controlling and providing nutrients such as fatty acids, amino acids and vitamins, which are required for immune cell differentiation and function, remains largely elusive. In this review, we summarize the current understanding of local nutritional exchange and control between immune and stromal cells in peripheral tissue and, when it is known, in the bone marrow. The parallels found between peripheral tissues and bone marrow stroma raises the question of how local metabolism is capable of influencing haematopoiesis and immunopoiesis. A better understanding of the local exchange of nutrients in the bone marrow can be used to improve immune cell formation during ageing, after haematopoietic stem cell transplantation and during immune challenge.

**Abbreviations**
ASCT2: alanine, serine, cysteine‐preferring transporter 2
ATP: adenosine triphosphate
BM: bone marrow
CD: cluster of differentiation
CXCL12: C‐X‐C motif chemokine ligand 12
CXCR4: C‐X‐C chemokine receptor type 4
CYP26: cytochrome P450 family 26
FAO: fatty acid oxidation
HSC: haematopoietic stem cell
ILC: innate lymphoid cell
IL: interleukin
MSC: mesenchymal stem cell
OXPHOS: oxidative phosphorylation
RA: retinoic acid
RAR*γ*: retinoic acid receptor *γ*
SLC: solute carrier family
SNAT: sodium‐coupled neutral amino acid transporters
TCA: tricarboxylic acid
Th: T helper
Tmem: memory T
Treg: regulatory T
VDR: vitamin D receptor

## Introduction

Immune responses are highly coordinated processes regulated on several levels by immune cells and a variety of non‐haematopoietic cell types. Non‐haematopoietic cells are commonly referred to as ‘stroma’ by haematologists and immunologists. Interactions with stromal cells are essential for immune cells at various stages of immune cell differentiation (immunopoiesis) and immune activation. The deletion of stromal cell types can therefore result in dysfunctional lymphoid organs and thereby hamper immune responses. In 1971, the bone marrow (BM) niche was described as a ‘haematopoietic inductive environment’ that supported the maturation of distinct immune cells.^1^ This early description of the niche was confirmed by the identification of distinct stromal niches for megakaryocyte and B lymphocyte differentiation in the BM.^2^, ^3^ In its function as a primary lymphoid organ, stromal cells form supportive microenvironments to maintain and regulate immune progenitors. Recent findings provide evidence that the BM serves as a secondary lymphoid organ containing many recirculating mature immune cell types of the innate and adaptive immune systems.

The BM stroma provides a supporting network of cells for the maintenance and survival of immune progenitors through direct cell–cell interactions and the paracrine release of cytokines. This local interaction of the BM stroma with immune cells enables them to respond efficiently to altered environmental cues. For example, during bacterial infections, the BM induces a strong myeloproliferative response through the increased stromal secretion of the pro‐inflammatory cytokine interleukin‐6 (IL‐6).^4^, ^5^

Interestingly, findings in recent years provide evidence for a local metabolite exchange and regulation of nutrients between the stroma and immune cells, which we refer to as ‘local metabolism’. The availability of micronutrients (e.g. vitamins) and energy sources such as amino acids and fatty acids are essential for immune cells. Differentiation and effector functions are metabolically tightly regulated. This link has given rise to a new field in immunology called immunometabolism.^6^ However, the impact of the BM stroma on differentiating and mature immune cells in regulating the local exchange and availability of nutrients is still an emerging field. In this review, we highlight recent discoveries how stromal cells use local metabolism of metabolites such as fatty acids, amino acids and vitamins to direct immune cell differentiation and function. Due to its moonlighting function as a primary and secondary lymphoid organ, we focus this review on the BM while extrapolating from studies that have identified regulation of immune cell metabolism by stroma in peripheral tissues.

## Stromal cytokine release regulates immune cell survival, differentiation and migration

The BM is arguably one of the most complex environments in the human body, merging four major biological systems: the skeletal system, the immune system, the endocrine system and the nervous system. This accumulation of biological systems gives the BM a unique capability to react immediately to external stimuli. Besides providing a niche for haematopoietic stem cells (HSC) and other haematopoietic progenitors, it contains the recently identified distinct niches involved in B‐cell lymphopoiesis, myelopoiesis and erythropoiesis. In addition, increasing evidence suggests that the BM hosts mature long‐lived immune cells such as memory T (Tmem) cells and plasma cells.^7^, ^8^, ^9^ Akin to immune progenitors, stromal components of the BM control mature immune cells through cell–cell interactions and the local release of cytokines.

The cells that constitute the BM niche have been extensively reviewed in Wei and Frenette.^10^ Quiescent HSC reside in the endosteal niche close to the bone, which is constituted predominantly of mesenchymal stem cells (MSC), bone‐forming osteoblasts and adipocytes. Early studies targeting specifically osteoblasts provided the first *in vivo* evidence for the existence of the haematopoietic niche by demonstrating that HSC frequency was controlled through cell‐extrinsic mechanisms.^11^, ^12^ Subsequent analysis revealed that many mesenchymally derived cell types including MSC and adipocytes contribute to the survival and regulation of HSC through secretion of major niche factors such as stem cell factor and the BM retention chemokine CXCL12.^13^, ^14^, ^15^ Differentiating HSC are found in the perivascular niche and associated with sinusoidal endothelial cells, CXCL12‐abundant reticular cells and MSC. The CXCL12‐abundant reticular cells were identified as a crucial stromal component in HSC and plasma cell maintenance as well as B‐lymphocyte differentiation by expressing high levels of CXCL12.^2^, ^16^ Associated with the vasculature, adrenergic nerve fibres control CXCL12 release from the BM stroma in an oscillating manner according to the circadian rhythm.^17^ This release is coordinated by noradrenaline from sympathetic nerves, which binds to *β*3‐adrenergic receptors on mesenchymal cells and subsequently controls the egress of haematopoietic progenitors from the BM.^18^ Aberrant norepinephrine transmission abrogates circadian secretion of CXCL12 from stromal cells.^18^

The release of cytokines such as stem cell factor and CXCL12 is crucial for the long‐term survival and function of the haematopoietic system. In this way, distinct BM niche environments control lineage commitment and immune cell egress. This knowledge has led to studies investigating whether co‐transplantation of niche cells improves HSC transplantation, showing that B‐cell recovery and engraftment can be improved in murine models.^19^ However, potentially due its complexity and technical challenges, current research has barely scratched the surface of the role of all BM constituents and their functions in various contexts. Especially their role in directing immune differentiation and their impact on immune cell metabolism remains poorly understood.

## Immunometabolism drives immune cell differentiation and function

Cellular metabolism balances energy‐producing processes by the degradation of macromolecules (catabolism) and energy‐consuming synthesis of new macromolecules (anabolism). During catabolic processes, adenosine triphosphate (ATP) is produced through two major pathways: glycolysis and oxidative phosphorylation (OXPHOS). Fast turnover of glucose to acetyl‐Coenzyme A during glycolysis makes ATP quickly available, but produces a lower energetic yield than the full degradation of carbon‐based macromolecules in the tricarboxylic acid (TCA) cycle. The latter process releases electron transporters nicotinamide adenine dinucleotide and flavin adenine dinucleotide, which are oxidized during OXPHOS in the mitochondria resulting in high ATP quantities. Amino acids and fatty acids can be used as alternative energy sources and enter the metabolic pathway in the TCA cycle driving glutaminolysis and fatty acid oxidation (FAO), respectively. Interestingly, many amino acid precursors derive from TCA intermediates and play a role in anabolic processes. Consequently, modulation of the glycolysis–OXPHOS balance can shift cellular bioenergetics and biosynthesis.

Immune cells have various metabolic demands based on their differentiation and function. Cells need to utilize different metabolic pathways in a time‐ and space‐dependent manner to produce the energy, metabolites and macromolecules required for their differentiation and function. An increasing body of evidence describes that the fine balance of OXPHOS and glycolysis is crucial for the differentiation of immune cells, including myeloid and T cells.^6^ In the cancer field, aerobic glycolysis, also known as Warburg effect, is a hallmark of cancer cell metabolism enabling them to proliferate extensively.^20^ Akin to cancer cells, the rapid expansion and proliferation of immune subsets during pathogenic challenges requires a similar shift to a glycolytic state.^21^ In contrast, quiescent cells use OXPHOS for self‐maintenance. It has only recently come to light that metabolic changes are indispensable for the induction of differentiation and not merely a bystander or consequence of it. In other biological tissues and systems, this question has already revealed that changes in metabolism result in differentiation.^22^ In the immunological context, neutrophil differentiation requires the metabolic shift from glycolysis to OXPHOS to terminally differentiate and exert its functions.^23^ Moreover, immune cell function can be altered by modification of cellular metabolism. Macrophage polarization is closely associated with a glycolytic M1‐phenotype and OXPHOS‐mediated metabolism of M2 macrophages.^24^ Preventing OXPHOS by blocking FAO in M2 macrophages led to decreased M2 activation and reduced parasite clearance.^25^ The importance of immune cell metabolism is not only restricted to the innate immune system, but has been shown to influence T‐cell fate. Pro‐inflammatory effector T cells such as T helper type 1 (Th1) cells and Th17 cells exhibit a glycolytic metabolism and are highly proliferative and short‐lived. In contrast, long‐lived Tmem cells and regulatory T (Treg) cells rely on FAO.^26^ This intrinsic metabolic difference was further confirmed by the switch of Th17 to Treg cells following inhibition of glycolysis using 2‐deoxyglucose.^27^ Keeping in mind that immune cells depend on an orchestrated sequence of metabolic events for lineage commitment, differentiation and functional outcome, we hypothesize that these metabolic pathways may, at least in part, be regulated by the surrounding stroma.

## Fatty acids in the bone marrow to support long‐lived immune cells

Fatty acid oxidation is an important metabolic and bioenergetic pathway for many immune cells. During this catabolic process, cytosolic fatty acids are degraded by the mitochondria, resulting in signalling intermediates and high yields of ATP, which promotes cell differentiation and self‐renewal. For example, in the gut, innate lymphoid type 2 cells (ILC2) are activated during helminth infection. Interestingly, deficiency of retinoic acid (RA) leads to an increase in ILC2 numbers and is primarily dependent on the uptake and catabolism of fatty acid from the environment.^28^, ^29^ Wilhelm *et al*. showed that this uptake was significantly increased in ILC2 from mesenteric adipose tissue,^29^ which in turn supports the FAO‐based metabolism in ILC2. This metabolic support is crucial during helminth infection as inhibition of FAO resulted in decreased ILC2 accumulation and cytokine production, leading to increased parasite burden.

Other lymphoid cells such as Treg cells are enriched in peripheral adipose tissues.^30^ Similar to ILC2, these cells strongly depend on FAO as their primary energy source independent of *de novo* fatty acid synthesis and mostly reliant on the import of fatty acids from the environment.^31^, ^32^, ^33^ Adipose Treg cells are induced upon several metabolic and environmental stimuli and have been suggested to control adipocyte function through a signal transducer and activator of transcription 6–phosphatase and tensin homologue axis.^34^ On the other hand, adipocytes can regulate T‐cell fate through major histocompatibility complex class II‐dependent secretion of interferon‐*γ*.^35^ However, the regulation of adipose‐derived metabolites of T‐cell differentiation remains largely unanswered.

Long‐lived immune cells such as Tmem cells and fetally derived B1a B cells seem to preferentially reside in fat tissues such as visceral fat depots and the peritoneum, respectively. These cell subsets depend on fatty acids as an energy source; they are even essential for their differentiation and maintenance.^36^, ^37^ The inhibition of glycolysis of CD8^+^ T cells using 2‐deoxyglucose results in an increased generation of Tmem cells, whereas the over‐expression of glycolytic genes prevent their formation.^38^ The necessary free fatty acids are synthesized upon IL‐7 stimulation from glycerol and consequently released from intracellular stores in CD8^+^ Tmem cell populations.^39^, ^40^ However, a recent study revealed that CD8^+^ tissue‐resident Tmem cells in the skin not only rely on FAO but also require exogenous fatty acids for their long‐term survival.^41^ Although the skin represents a lipid‐rich environment, the support of fatty acids from different stromal cells requires further investigation. Tmem cells are found in several adipose tissues where they serve as a reservoir and ameliorate recall responses compared with peripheral Tmem cells. Intriguingly, Tmem cells from adipose tissue are metabolically distinct and show a higher rate of exogenous lipid uptake and mitochondrial respiration than Tmem from the spleen or lamina propria.^42^ Following re‐challenge, mesenteric Tmem cells immediately increase pro‐inflammatory cytokine production whereas splenic Tmem cells are less reactive.

In the past two decades, increasing evidence supports the notion that Tmem cells can be found in distinct niches in the BM.^7^, ^43^, ^44^ Interestingly, redirecting T cells to the BM by over‐expressing C‐X‐C chemokine receptor type 4 (CXCR4) promotes Tmem cell formation and improves effector expansion upon recall.^45^ The adult human BM contains > 70% adipocytes. The environmental parallel to the peripheral Tmem cells in adipose tissues allows intriguing analogies. However, BM adipocytes are of distinct origin in comparison to peripheral white or brown adipocyte tissue.^46^, ^47^ Although similar to peripheral tissues, the fatty environment of the BM is home to Treg cells, which have been described to take an active part in the BM niche function. These long‐lived cells migrate into the BM through the CXCL12/CXCR4 axis forming a Treg cell reservoir.^48^ In the BM, Treg cells have been shown not only to regulate the niche compartment – and thereby influence B‐cell differentiation – but also to support maintenance of long‐lived plasma cells.^49^, ^50^ Interestingly, a recent report suggests that niche Treg cells may be distinct from peripheral Treg cells by their high expression of the HSC marker CD150 and their capacity to release adenosine into their local environment, which in turn reduces oxidative stress in HSC.^51^ However, studies so far have not investigated whether the BM stroma contributes to Treg cell metabolism by provision of free fatty acids as described in peripheral tissues.

Increased marrow adiposity has been associated with increased myelopoiesis in the BM.^52^ The significance of marrow adipocytes in myelopoiesis was highlighted when adipocytes were depleted from the BM niche in an acute myeloid leukaemia model, leading to decreased erythroid and myeloid lineages.^53^ Furthermore, a high‐fat diet is known to increase BM adiposity and skew immune cell differentiation towards myeloid lineage. In their seminal study, Luo *et al*. fed mice with a high‐fat diet for several weeks, leading to the preferential differentiation of MSC into adipocytes through the activation of the adipogenic transcription factor peroxisome proliferator‐activated receptor *γ*2. This resulted in a decreased number of haematopoietic progenitor populations and increased myelopoiesis.^54^ Even more intriguing was the finding that antibiotic treatment alleviated the high‐fat diet‐mediated effect on the BM stroma. The impact of the intestinal microbiota on the BM is becoming increasingly evident. Small microbially derived metabolites called short‐chain fatty acids modify osteoclast differentiation by shifting their metabolism from OXPHOS to glycolysis.^55^ These metabolic impacts from systemic environmental changes may modify different niche constituents and translate into aberrant immune differentiation and function. The precise mechanism on how BM adipocytes are involved in shaping the immune response is less well defined. However, some cells of the myeloid compartment such as neutrophils rely on FAO for their differentiation.^23^ This fact allows the hypothesis of metabolic support of marrow adipocytes in lineage commitment, differentiation and maintenance. In line with this, active fatty acid transfer from stromal cells was shown to be an energy source fuelling the metabolism of a variety of cancers. For example, ovarian cancer metastasis primarily manifests in omental adipose tissue, thereby using the adipocyte‐derived fatty acids to support their growth.^56^ Similar mechanisms of lipid transfer are likely to be present in the BM. Evidence from acute myeloid leukaemia suggests that cancer cells induce lipolysis in BM adipocytes and sequester these liberated lipids.^57^

**Figure 1:**
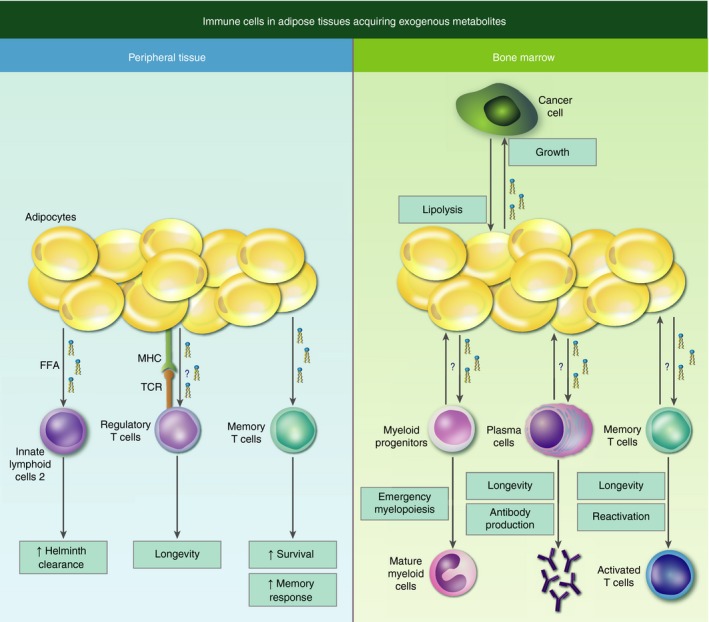
Concept of immune cells in adipose tissues acquiring exogenous metabolites to support their maintenance, differentiation and function. Long‐lived immune cells in peripheral tissues and in the bone marrow rely on faty acid oxidation (FAO). In peripheral tissue this metabolic programme is supported by adipocytes through the release of fatty acids. Akin to this concept, cancer cells stimulate lipolysis in bone marrow adipocytes to fuel their energy demands. If this local exchange of fatty acids between bone marrow adipocytes and differentiating (e.g. myeloid cells) and residing immune cells (e.g. plasma cells, memory T cells) is occurring under normal conditions remains to be answered.

In conclusion, adipocytes in peripheral tissues are capable of controlling long‐lived memory cell responses by providing essential metabolic support. Similar mechanisms may exist in the BM but have not been addressed in immune homeostasis. Recent examples from the cancer field provide compelling insights into the means of communication at a metabolic level between immune cells and adipocytes (Fig. 1). Whereas many of these studies have focused on the bioenergetics linked to lipid‐based metabolism, exogenous lipids may also be implicated in membrane formation or as signalling mediators.^32^ With regards to the vast numbers of adipocytes in the adult BM, a careful re‐evaluation of the metabolic support of BM adipocytes may provide better understanding in shaping immunometabolism in this compartment.

## Amino acids, a keystone for BM and immune cell differentiation and function

In the last few years, amino acids and their transporters have emerged as key regulators of haematopoiesis and immunopoiesis. A growing number of studies show the importance of nutrient uptake for cell survival, proliferation, differentiation and immunity. While the role of amino acids for cell survival and proliferation is not surprising, their importance for differentiation and immune responses is less obvious.

Pioneering studies from Kornberg *et al*. in the mid‐1940s showed that rats fed with a low‐protein diet developed a severe granulocytopenia and anaemia.^58^, ^59^ Interestingly, granulopoiesis and erythropoiesis were restored with a mixture of essential amino acids or with a combination of folic acid (vitamin B9) and casein. This was the first evidence for a role of amino acids for cell differentiation beyond cell survival. Seventy years after Kornberg's initial study, technology and science had advanced enough to define which amino acids are essential for HSC fate and function. Taya *et al*. 60 showed that the concentration of all 20 amino acids was over 100 times higher in the BM compared with peripheral blood. Depletion of dietary valine led to a drastic and selective loss of the HSC population. Moreover, vascular epithelial cells and cells expressing the MSC‐marker platelet‐derived growth factor receptor, both part of the HSC niche, secrete some amino acids, specifically highlighting these BM cells as putative regulators of local amino acid concentrations in the BM niche. However, the molecular mechanism for valine in HSC survival and self‐renewal is still unclear.

In contrast to valine, glutamine metabolism is well studied and is an essential part of erythropoiesis. The expression of the main glutamine transporter solute carrier family 1A5 (SLC1A5) [also called alanine, serine, cysteine‐preferring transporter 2 (ASCT2)] as well as glutamine‐driven metabolism for ATP and nucleotide production is crucial to maintain erythropoiesis.^61^ Consequently, preventing glutamine uptake halts HSC differentiation at an immature myeloid state even in the presence of the relevant cytokines for erythropoiesis (e.g. erythropoietin). This finding identifies glutamine, a few decades after Kornberg's initial studies, as the critical amino acid for erythroid differentiation. Besides its importance in erythropoiesis, glutamine exerts pleiotropic functions during T‐cell activation and differentiation. Naive quiescent T cells rely on the TCA cycle and OXPHOS to produce ATP. Once activated, a metabolic switch occurs and T cells increase glycolysis, fatty acid synthesis and glutaminolysis.^62^ Activated T cells strongly induce expression of the main glutamine transporters *SLC38A1*,* SLC38A2* and *SLC1A5* [sodium‐coupled neutral amino acid transporters 1 and 2 (SNAT1, SNAT2) and ASCT2, respectively].^63^, ^64^ In line with this, activated T cells have up to 10‐fold higher glutamine uptake than quiescent T cells, and blocking glutamine uptake impairs T‐cell homeostasis and differentiation. Mice deficient in ASCT2 have reduced numbers of CD4^+^ T and Tmem cells compared with wild‐type mice, whereas CD8^+^ T and Treg cell populations remain unaffected.^64^ CD4^+^ T cells from ASCT2^−/−^ mice express activation markers such as CD69 or CD25 but are unable to raise an appropriate Th1 or Th17 immune response. Interestingly, IL‐2 production is not affected. These results demonstrate that glutamine is required for CD4^+^ T‐cell homeostasis, differentiation and function.

Amino acid consumption affects immunity in various, often opposite, ways – like arginine, which is able to enhance macrophage cytotoxicity but blocks Th1 and Th17 responses. Arginine is metabolized in macrophages to produce nitric oxide and citrulline by inducible nitric oxide synthase, and the polyamine precursors l‐ornithine and urea by arginase I and II. These molecules are crucial for the cytotoxic functions of macrophages, cell proliferation and antibacterial response.^65^ Interestingly, T cells and macrophages can modulate reciprocal immune outcomes via metabolites. For example, expression of inducible nitric oxide synthase and arginase I is regulated by Th1 and Th2 cytokines, respectively.^66^ Macrophages activated by the Th2 cytokines IL‐4 and IL‐13 highly express arginine transporter SLC7A2 (also named CAT2) and arginase I and induce depletion of arginine from their local environment.^67^ This change in local arginine concentration ultimately decreases CD3*ζ* expression in activated T cells and diminishes their proliferation.^67^ The same deprivation can be observed in several types of cancers with a similar effect on T‐cell immunity. Tumour‐associated myeloid cells (referred to as myeloid suppressor cells) consume large amounts of arginine in various cancer types and consequently block anti‐tumour effects of infiltrating T cells.^68^, ^69^ The metabolic interaction between cancer cells and their stroma is even more interlinked. For example, pancreatic cancer cells increase amino acid uptake to proliferate through stimulation of alanine secretion from stromal cells in their microenvironment.^70^ Based on Taya *et al*.'s findings, similar paracrine mechanisms may be present in the BM mediated by platelet‐derived growth factor receptor‐positive cells in a physiological context.

**Figure 2:**
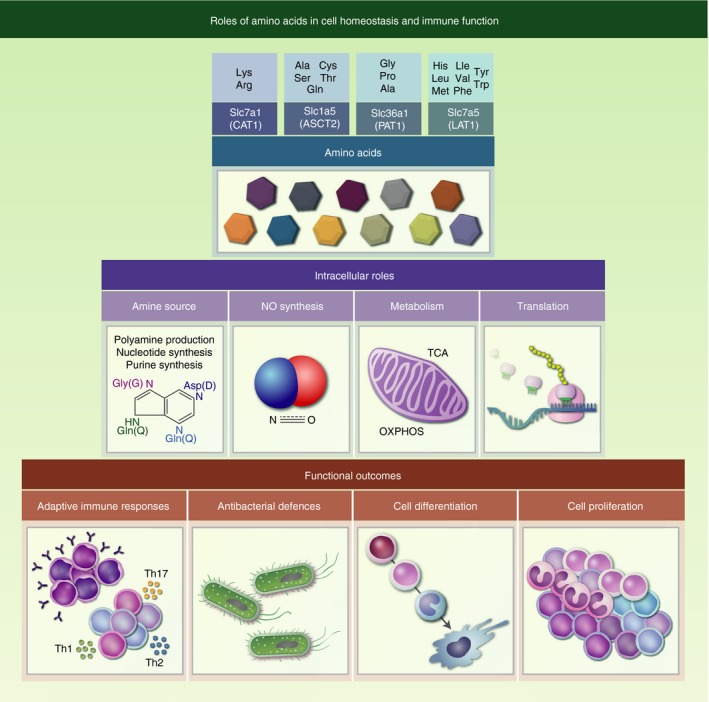
Roles of amino acids in cell homeostasis and immune function. Exogenous uptake of amino acids is controlled by the expression of the SLC transporter family. Members of the LAT protein family (here: SLC7A5) form a heterodimer with SLC3A2 for their functional expression. The acquired pool of amino acids can serve several purposes besides its primary role in translation, amino acids are also the main source of intracellular amine to synthesize purines/nucleotides to ensure DNA stability and produce nitric oxide to regulate immune responses. Furthermore, amino acid‐mediated changes in metabolism and increased ATP production drive cell proliferation, cell differentiation and in some cases can maintain cell quiescence.

Over the past years, our understanding of amino acid catabolism and their various roles in homeostasis and immunity has exponentially increased. Distinct amino acids are crucial for cell survival, proliferation or differentiation (Fig. 2). However, even if increasing evidence highlights that local amino acid metabolic interaction influences cell behaviour in various ways, there is still a lot to discover about the specificities and roles of amino acid metabolic pathways in stromal and immune cells.

## Vitamin availability affects immune cell outcome in periphery and the BM

Some micronutrients such as vitamins are not used for the production of ATP or macromolecules but regulate many metabolic pathways. The term ‘vitamin’ classically refers to organic compounds, which cannot be synthesized in sufficient amounts, but are essential to the health of an organism. Vitamins have pleiotropic effects in humans, some of which are rather unspecific such antioxidants, whereas others have more specific functions as enzymatic co‐factors or hormones. The importance of vitamins in the immune system has been known for a long time.^71^ Retinoic acid, more commonly known as vitamin A, is essential for a functional immune system. The amount of RA in the gut is controlled by intestinal epithelial cells, stromal cells and a distinct subset of intestinal dendritic cells.^72^, ^73^, ^74^ Interestingly, dendritic cells store RA intracellularly and its coordinated release controls T‐cell activation.^75^ This is of particular interest as RA promotes the generation of anti‐inflammatory Treg cells, at the expense of pro‐inflammatory Th17 differentiation.^74^, ^76^ This is further supported by experiments using human dendritic cells, which when primed with RA promote Treg cell differentiation *in vitro*.^77^ The control of local concentration of RA appears to be crucial for adaptive immunity in the gut. Conversely, lower and potentially more physiological concentrations of RA in the gut are essential for Th17 development.^78^ These studies illustrate the delicate control of local RA concentrations in the gut and their crucial impact on T‐cell fate.

Apart from the gut stroma, vitamin A also plays an important role in the BM. Activation of RA signalling preferentially promotes HSC dormancy in the BM and so controls HSC exhaustion. When RA is limited via dietary uptake, HSC are activated to proliferate and differentiate.^79^ This is in line with findings that loss of the RA receptor retinoic acid receptor *γ* (RAR*γ*) leads to decreased HSC number and increased differentiation.^80^ The same study also showed that RAR*γ* activation increases the self‐renewing capacity of HSC.^80^ Besides regulating HSC dormancy directly, the BM stroma actively participates in the control of and response to RA levels. The loss of RAR*γ* in the BM stroma induces a myeloproliferative syndrome, which was long believed to be caused by cell‐intrinsic effects only. However, transplantation of wild‐type HSC into an RAR*γ*‐deficient microenvironment was sufficient to initiate myeloproliferative syndrome.^81^ Interestingly, the differentiation of neutrophils can also be blocked at the promyelocyte stage, when cells lack functional RA signalling. As a result, RA has been used to promote terminal differentiation of promyelocytes in acute promyelocytic leukaemia.^82^, ^83^ The BM sequesters significant amounts of the RA precursor retinol, even during dietary restriction of vitamin A.^84^ This may be a compensatory mechanism of the BM to assure normal haematopoiesis. In addition to its sensitivity to RA, the BM stroma is capable of controlling local concentrations of RA through expression of the RA‐degrading enzyme cytochrome P450 family 26 (CYP26), a member of the cytochrome P450 family. The authors found that limiting RA levels by CYP26‐expressing cells in the BM niche favours HSC proliferation.^85^ These CYP26‐expressing stromal cells may play an important role in providing an acute promyelocytic leukaemia‐permissive BM environment.^86^ The exact role of RA and the control of its local concentration on immune cell differentiation remain to be further explored by dissecting the different niche compartments for granulopoiesis and HSC maintenance.

Cholecalciferol (or vitamin D3) is predominantly synthesized in the skin from cholesterol through ultraviolet B radiation. As a result of the dependency on sun exposure, vitamin D levels depend on the geographical location of individuals and may require dietary supplementation. Dietary or synthesized vitamin D precursor cholecalciferol is converted in the liver to calcifediol and consequently hydroxylated into its bioactive form 1,25‐dihydroxyvitamin D. Vitamin D controls calcium metabolism, so vitamin D deficiency can lead to osteomalacia, a softening of the bone tissue. Lack of 1,25‐dihydroxyvitamin D3 is implicated in alteration of immune function and can lead to autoimmune diseases such systemic lupus erythematosus and rheumatoid arthritis.^71^ Vitamin D controls the immune system by exerting an anti‐inflammatory effect on lymphocytes leading to decreased T‐cell proliferation through IL‐2 suppression and decreased antibody production in B lymphocytes.^87^, ^88^ In addition, it tips the Th1–Th2 balance towards anti‐inflammatory Th2 cells through repressing interferon‐*γ* secretion and promoting IL‐4 production, which is crucial for Th2 development.^89^ This was further confirmed in mice deficient for the vitamin D receptor (VDR) describing normal lymphocyte composition, but reduced response to anti‐CD3 T‐cell stimulation.^90^ Further, this study showed that immune alteration in VDR‐knockout mice is rescued by normalization of calcium metabolism. Although the loss of VDR in non‐haematopoietic cells did not change HSC frequency in the BM niche, it increased splenic haematopoiesis.^91^ Interestingly, vitamin D directly impacts on haematopoietic stem and progenitor cells in zebra fish by increasing their proliferation, independent of changes in calcium metabolism.^92^ *In vitro*, adipogenic differentiation of BM‐derived MSC is inhibited in a VDR‐dependent manner suggesting a mechanism to control BM cell composition.

Vitamin availability impacts on immune cell function in peripheral tissues and the BM. For vitamin A, we are beginning to understand that niche cells are capable of controlling and providing vitamins in a coordinated manner to immune cells. The resulting impact of vitamins on immune cell metabolism is part of current research efforts. Vitamin D was shown to promote tolerogenic dendritic cell functions by favouring OXPHOS, fuelling it with glycolytic pyruvate.^93^ This is in line with recent findings of increased expression of genes involved in OXPHOS, TCA cycle and ATP synthesis upon vitamin D stimulation of myeloid cells.^94^ These data underline the importance of vitamins for the immune system and their potential to induce metabolic changes. In addition, several vitamins act as co‐factors for metabolic enzymes, making them attractive targets for further investigations.^95^ More studies will be required to investigate the role of vitamins in shaping cellular metabolism during immunopoiesis.

## Perspectives

The field of immunometabolism has provided strong evidence for the close link between cellular metabolism and immune cell differentiation and function. The switch between distinct metabolic pathways maintains energy homeostasis and produces metabolites and intermediates required for the molecular adaptation. Despite the identification of metabolic requirements in a variety of immune cells, it remains unclear how the decision for specific metabolic pathways is established. Nutrients are sequestered by many stromal cells and released upon physiological cues. In particular, well‐controlled immunological niches such as the BM, which contain a distinct stromal composition, may have means to control and provide nutrients to developing and resting immune cell populations and thereby shaping their cellular metabolism. Evidence for the importance of nutrient support by the haematopoietic BM niche is emerging. For example, the availability of vitamins is closely controlled by stromal cell in the BM niche and thereby impacts on lineage commitment and stem cell maintenance. However, despite our knowledge that many vitamins are important co‐factors for metabolic processes, we are only now starting to explore the impact of vitamins in reshaping immune cell metabolism. Other energy‐rich nutrients such as fatty acids and amino acids are released in a controlled manner during times of high energy demand, though the importance of this process in the BM remains largely neglected. Few explorative studies in the cancer field suggest local metabolite exchange in the BM. However, despite these findings, many open questions in the local metabolic interactions of BM stroma and immune cells remain (see Box 1). In addition to nutrient availability, local oxygen levels must be taken into account when investigating cellular metabolic decisions. Low oxygen levels promote the switch to glycolysis through the transcription factor hypoxia inducible factor‐1.^96^, ^97^ Interestingly, effector memory CD4^+^ T cells are highly migratory and travel between oxygenated blood and hypoxic peripheral tissues. These cells are characterized by an increased spare respiratory capacity to balance their energy levels even under hypoxic conditions compared with naive CD4^+^ T cells.^98^ The local oxygen levels in the BM were thought to be low, providing an explanation for the glycolytic state of HSC but this remains controversial and makes the interpretation of microenvironmental oxygen conditions in the BM difficult.^99^

Box 1 **Open questions regarding the nutritional support of immune cells**

1. Are adipocyte‐derived fatty acids required to support the fatty acid oxidation‐based metabolism in immune cells during maintenance and differentiation in the bone marrow?
2. Can stroma‐derived amino acids support immunopoiesis? If yes, via which mechanisms?
3. Does vitamin availability in the bone marrow direct immunometabolism of differentiating immune cells?
4. Could ‘local metabolism’ be a drug target to improve lineage bias during ageing and haematopoietic stem cell transplantation?

A major challenge to address these questions remains the limitations of tools and technologies in order to study local metabolism *in vivo*. Using *in vitro* culture systems, cell interactions can be studied in controlled environments, but these often provide a narrow insight into complex biological systems. Technological advances in BM imaging such as high‐resolution bone microscopy and intravital imaging are providing novel tools to visualize and study distinct niche environments.^100^, ^101^ Furthermore, transcriptomic and proteomic analysis of single BM stromal cells will further improve our understanding of distinct stromal cell components in the BM and highlight the differences with peripheral tissues.^102^ This information will be crucial to improve modelling of the BM niche *in vitro* and improve *ex vivo* systems such as ‘BM‐on‐the‐chip’ allowing modelling of the complex marrow environment.^103^, ^104^ Lastly, conditional genetic deletions targeting crucial genes involved in metabolic nutrient exchange can serve as powerful tools to identify stromal subsets involved in the control of immunopoiesis and the maintenance of long‐lived cells in the BM.

Addressing the issue of local metabolism in the BM will give us a better understanding of immune cell formation and consequently be useful for fields addressing aberrant immunopoiesis during ageing, metabolic disorders and haematopoietic stem cell transplantation.

## Disclosures

The authors declare no competing financial interests.
